# Extinction Coefficients of NO_2_ and N_2_O_4_*

**DOI:** 10.6028/jres.080A.017

**Published:** 1976-04-01

**Authors:** Arnold M. Bass, Albert E. Ledford, Allan H. Laufer

**Affiliations:** Institute for Materials Research, National Bureau of Standards, Washington, D.C. 20434

**Keywords:** Absorption, extinction coefficients, N_2_O_4_, NO_2_, spectra, temperature effects

## Abstract

The extinction coefficient of NO_2_ has been measured in the spectral range 185 to 410 nm as a function of temperature between 235 and 298 K. In order to correct for the effect of the dimer absorption, the extinction coefficient of N_2_O_4_ has also been measured. The effect of a decrease in temperature upon the NO_2_ absorption is a reduction in the extinction coefficient of approximately 10 percent in the range 320 to 380 nm.

## 1. Introduction

The absorption of solar radiation by NO_2_ is of prime importance in the chemical processes which may occur in the troposphere and stratosphere. The photo-dissociation of NO_2_ is an important source of oxygen O(^3^P) atoms in the atmosphere. At all wavelengths shorter than the dissociation limit, 398 nm, absorption of light by NO_2_ results in dissociation of the molecule with the formation of an oxygen atom. The fate of the O atom, ultimately O(^3^P) in large part, is to react with O_2_ resulting in O_3_, the chemistry and physics of which are crucial to the dynamics of the stratosphere. A determination of the atom yield in the atmosphere depends on a knowledge of the NO_2_ absorption cross-section, concentration and quantum yield of dissociation, and the solar flux over the wavelength range 185–410 nm.

The measurements on NO_2_ reported in the previously published literature have been made at room temperature. Considerations of the photochemistry of NO_2_ in the stratosphere require a knowledge of the extinction coefficients at ambient stratospheric conditions —low temperature and low pressure. Extrapolation of data obtained at room temperature to stratospheric temperature requires a knowledge of the states involved in the absorption. If any of the absorption is attributable to “hot-bands” the relative importance of such absorption will decrease markedly with decreasing temperature. Since the details of the transitions involved in the NO_2_ absorption spectrum over this wavelength range are not entirely understood, we have undertaken to determine the experimental behavior of the extinction coefficient as a function of temperature.

The measurement of the extinction coefficient of NO_2_ at any temperature is severely complicated by the presence of its dimer, N_2_O_4_. The equilibrium mole fraction of N_2_O_4_ is a function of both temperature and pressure. It is well known that low pressures minimize the N_2_O_4_ contribution. However, as the temperature of a mixture is reduced, the mole fraction of N_2_O_4_ increases. It is a relatively simple matter to determine the concentration ratio (NO_2_/N_2_O_4_) based upon thermodynamic considerations [1].^1^ While concentration corrections to the measured absorption may be small, a possibly greater error would result from the absorption by N_2_O_4_ if the extinction coefficient of N_2_O_4_ is large compared to that of NO_2_. It was imperative, therefore, to measure the extinction coefficient of N_2_O_4_.

Hall and Blacet [2] have reported values of the extinction coefficients for N_2_O_4_. Their experiments, however, were not performed under conditions where N_2_O_4_ formation was favored, i.e., low temperature and high pressure.

Three previous photoelectric measurements of the ultraviolet absorption cross-sections for NO_2_ have been reported. Nakayama et al. [3] examined the region from 108–270 nm with a spectral resolution of 0.02 nm. Corrections for the overlapping absorption by the dimer, N_2_O_4_, were made at five selected wavelengths between 190 and 240 nm by making measurements at several pressures, and extrapolating to zero pressure. Hall and Blacet [2] made measurements from 240–500 nm with an average spectral band width of 0.4 nm on three mixtures each of which contained appreciable N_2_O_4_. By using the equilibrium expression of Verhoek and Daniels [4] and Beer’s law, they were able to determine the extinction coefficients for both NO_2_ and N_2_O_4_. Johnston and Graham [5], in connection with a study of the absorption by nitric acid vapor, determined the NO_2_ extinction coefficient at low pressures and with a long optical path over the wavelength range 185–420 nm. Because of the low pressures used, the N_2_O_4_ was negligible.

## 2. Experimental Detail

All measurements were made with a 0.75 m Fastie-Ebert monochromator equipped with a 2400 groove/mm grating. With 10 *μ* slits, the spectral resolution was 0.01 nm. The actual measurements were made at intervals of 0.125 nm with a spectral resolution of 0.015 to 0.04 nm. The light source for the absorption studies was either a low-pressure hydrogen discharge for the region 185–360 nm, or, in the region above 360 nm, a quartz-iodine incandescent lamp. The entire gas handling system was fabricated of stainless steel and monel to minimize surface decomposition of the NO_2_. Pressures were measured by means of a capacitance manometer.

### Experimental: N_2_O_4_

**Figure 1:**
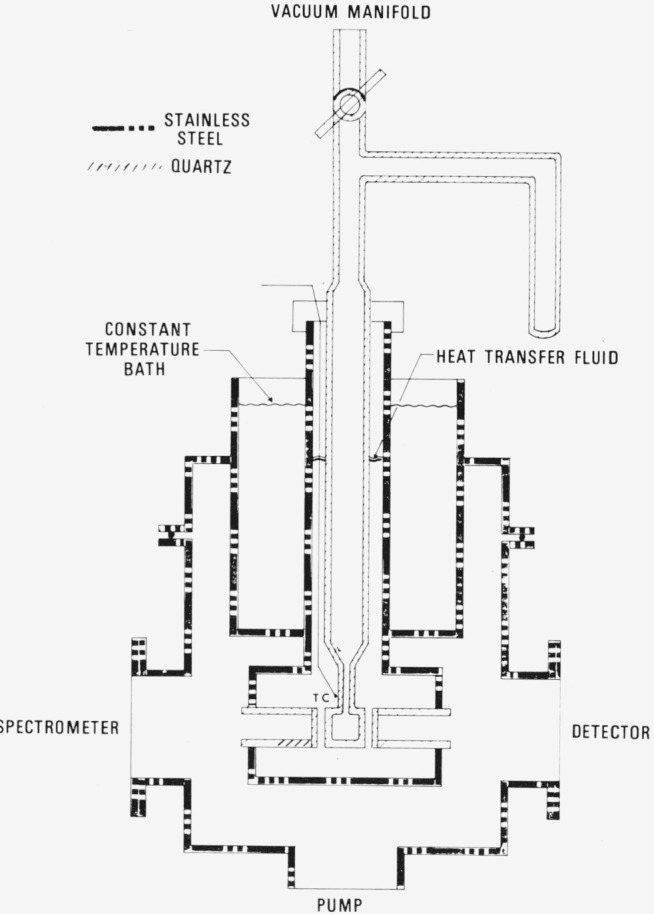
Cell used to measure ϵ (*N*_2_*O*_4_).

Two distinct, separate measurements were made; one involving the spectrum of N_2_O_4_ and the other of NO_2_. The spectrum of the former was obtained by using a low-temperature cell which has been previously described [6]. A Pyrex^2^ tube was sealed to one of several fused silica absorption cells of path lengths between 0.1 mm and 5.0 mm. The Pyrex portion was equipped with a side-arm which could be used as a cold trap. Purified NO_2_ was distilled into the cell through polytetrafluoroethylene vacuum valves. This cell was immersed in another cell fabricated of stainless steel, shown in figure 1. The heat transfer fluid was either methanol or 2,2,2-trifluoroethanol, both of which possess excellent light transmission properties at wavelengths as low as 185 nm at low temperature. Constant temperature was attained using either *n*-pentanoic acid slush (240 K) or CCl_4_ slush (250 K). At these temperatures and at the pressures used, the NO_2_ – N_2_O_4_ equilibrium mixture remains gaseous. The absorption cell temperature was measured with a chromel-constantan thermocouple which was in contact with the cell. As may be seen in figure 1, the surrounding volume was evacuated thereby eliminating the problem of frost formation on the fused silica windows.

In a typical experiment, the transmitted light intensity was determined through both the cooled absorption cell and optical system. Then NO_2_ was condensed into the side-arm of the absorption cell. Removal of the cold trap permitted the NO_2_ – N_2_O_4_ mixture to vaporize once again and fill the entire cold absorption cell. The material was allowed to equilibrate thermally and then the transmitted intensity was measured. The system was assumed to be in equilibrium when the measured absorption at any given wavelength remained invariant with time.

**Figure 2:**
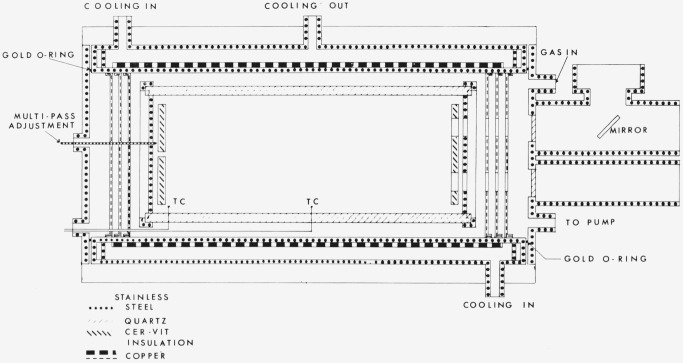
Multiple-pass low-temperature cell.

To describe adequately the ratio of NO_2_/N_2_O_4_, determination of both the temperature and pressure in the absorption cell are required. The temperature determination is straightforward. The pressure in the absorption cell could not be monitored during the course of an experiment (see fig. 2). Furthermore, portions of the cell were usually at two different temperatures during any particular measurement, i.e., room and some reduced temperature. As a consequence of these two factors, the pressure and hence the concentration of both monomer and dimer in the cold portion of the cell was determined by calculation based upon the conservation of N atoms in the system. The details of the calculation are shown in appendix A. The calculation does require knowledge of the temperature variation of *K_p_* which was derived from Chao et al. [1], and is partially described in appendix B.

### Experimental: NO_2_

In order to minimize the effect of the N_2_O_4_; the NO_2_ absorption measurements required low pressures and, therefore, long path lengths for which a variable temperature stainless steel cell was constructed (fig. 2). The cell was approximately 50 cm long and by using the multiple-pass design of White [7a] and Bernstein and Herzberg [7b] path lengths up to a maximum of 10 meters could be used. Since the cell was also to be used at low temperatures, the multiple-reflection mirrors were connected to each other by rigid fused silica rods to insure that the path length between the mirrors remained fixed as the cell temperature was changed. The arrangement also permitted adjustment of the optics on the bench and insertion, as a unit, into the cell.

The ends of the cell were thermally isolated from the environment by gold-plated copper radiation shields. The multiple-reflection mirrors were made of a low thermal expansion material and were aluminized and overcoated with magnesium fluoride. Temperature control was obtained by circulation of a refrigerated fluid, usually methanol, through the outer jacket of the cell. Further, the outer surface of the cell was coated with copper using a “flame-spraying” process to further insure uniformity of temperature along the cell. The gas sample temperature was measured by means of three calibrated chromel-constantan thermocouples inside the cell. At a cell temperature of 220 K the temperature variation of the sample over the length of the cell was approximately 1 °C.

Care was taken to avoid the use, as far as possible, of any materials which would be subject to corrosion by NO_2_. The vacuum seals were made by compressed gold O-rings. Two layers of aluminized-mylar were wrapped around the outer shell to provide insulation of the cell from room temperature.

The absorption cells were placed in the exit beam of the monochromator. Immediately in front of the cell, a sapphire plate was used to split the light beam so that a portion of the signal illuminated a 13-stage photomultiplier tube. The signal measured by this photomultiplier monitored the variation of the light source. Corrections for changes in the incident light signal were applied in the data reduction process. A second photomultiplier tube recorded the light flux transmitted through the absorption cell.

In all experiments, data acquisition was automated by photon counting equipment in conjunction with a stepping motor control for the monochromator wavelength drive. The operation of the equipment has been previously described [8].

The procedure used in a typical NO_2_ measurement involved a scan over the wavelength region to be examined, with the cell evacuated. The ratio of the incident signal to the transmitted signal (through the multpile-reflection cell) as a function of wavelength was determined. Then the cell was filled with a known pressure of NO_2_ as measured with a capacitance manometer and the scan repeated. The data were reduced by computer calculation after corrections were made for both the concentration and absorption due to N_2_O_4_.

The NO_2_ was obtained commercially and purified by reaction with excess O_2_. When cooled to −78 °C, a pure white solid was obtained which, following thorough pumping, was warmed, distilled through P_2_O_5_ and subsequently stored in the dark in a glass bulb. NO, a probable impurity, was absent in a 1 torr 3 sample of NO_2_ as indicated by the absence of absorption of the strong (A − X) system at 226 nm. A minimum of 0.005 torr NO would be observable under these conditions. To minimize errors in the determination of the extinction coefficients of both N_2_O_4_ and NO_2_, the following procedure was followed. Initial measurements were performed at low pressure over the complete wavelength region. Under these conditions, the concentration of N_2_O_4_ was minimal and a correction due to its concentration, but not its absorption, could be made. The values obtained are an upper limit for *ϵ*NO_2_ since some of the absorption is undoubtedly due to N_2_O_4_. The next series of experiments involved measurement of the N_2_O_4_ absorption as previously described. Here again, a correction due to the presence of NO_2_ could be made; both as to its concentration and absorption. In the region where the relative values of *ϵ*(NO_2_) and *ϵ*(N_2_O_4_) are about equal, the correction is less than 1%. At shorter wavelengths, where *ϵ*(N_2_O_4_) ≫ *ϵ*(NO_2_), an error in the latter has but a small effect on the determination of the former. With an adequate determination of *ϵ*(N_2_O_4_), an accurate value of *ϵ*(NO_2_) was readily obtainable.

## 3. Results and Discussion

### 3.1. N_2_O_4_

The extinction coefficient *ϵ* is defined by the Lambert-Beer equation: *I*/*I*_0_ = exp (−*ϵpx*) where *I* and *I*_0_ are the transmitted and incident light intensities, *p* the pressure in atmospheres at 273 K,^4^ and *x* the path length in cm.

The N_2_O_4_ absorption measurements were performed at −23 °C at pressures of about 30 torr and at room temperature at high pressures (117–500 torr). Under these conditions, the mole fraction of N_2_O_4_ represents between 40–80 percent of the sample. The actual experimental conditions are shown in table 1.

**Figure 3:**
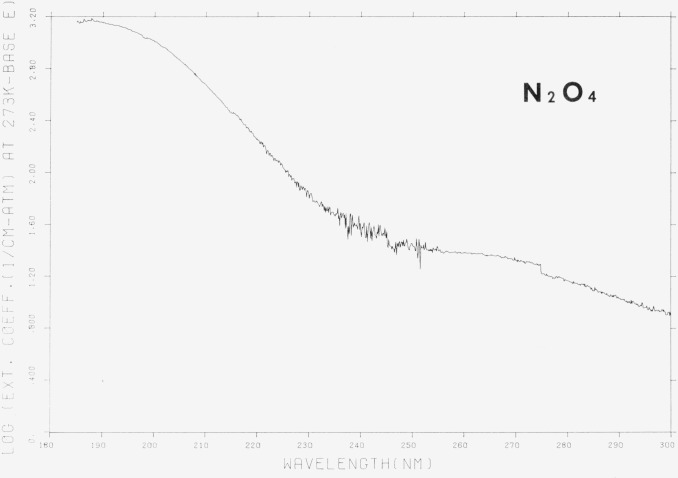
Common logarithm of the extinction coefficients of *N*_2_*O*_4_, 180–300 nm, ϵ in cm^−1^ (atm at 273 K) ^−1^ base e. [Plot is a computer reconstruction of averaged data. See text.]

**Figure 4:**
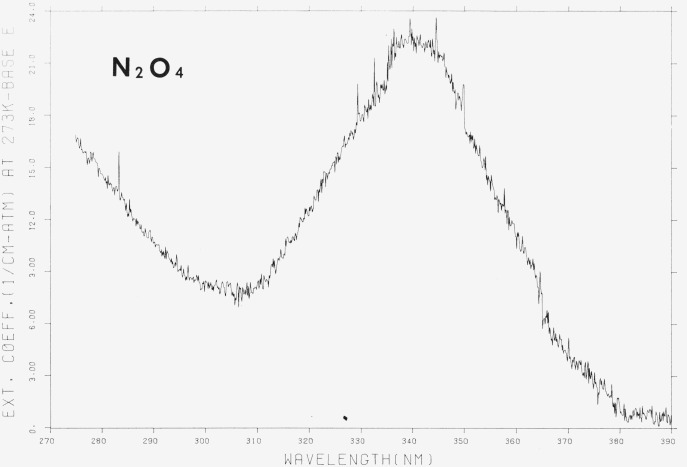
The extinction coefficients of *N*_2_*O*_4_, 270–390 nm, ϵ in cm^−1^ (atm at 273 K) ^−1^ base e. [Plot is a computer reconstruction of averaged data. See text.]

The results, which are shown in figures 3 and 4 and in table 3, were obtained at low and room temperature but have been corrected to the equivalent pressure at 273 K and represent the non-weighted average of at least 2 and usually 3 values at each wavelength. No temperature effect on the spectrum was observed. The data, then, represent the extinction coefficient of N_2_O_4_. There have been only two reported examinations of the N_2_O_4_ spectrum with which our results may be compared. In the shorter wavelength region, (185–240 nm) an approximate value for *ϵ*(N_2_O_4_) = 950 atm^−1^ cm^−1^ at 197 nm has been reported as the maximum value [3] which is to be compared to our value of ~ 1180 atm^−1^ cm^−1^ at 197 nm. Since the previous work is, in reality, an estimate and was obtained in systems with low N_2_O_4_ concentrations (~ 3 percent in measurements at 195 nm), the discrepancy of 20 percent is small. Of greater significance is the observation that the maximum absorption appears in the present work not at 197 nm, but at ~ 190 nm. Any rationale for the discrepancy would, of course, be speculative, but it should perhaps be noted that the values for *ϵ* (NO_2_) determined by Nakayama et al. [3], in the region below 200 nm are significantly greater than determined in the present work. It is possible that the absorption, incorrectly attributed to NO_2_, was in fact due to N_2_O_4_ whose concentration could have been incorrectly determined.

The N_2_O_4_ data with which we have the widest correspondence are those of Hall and Blacet [2]. The results of both sets of data have the same general shape with the first maximum at ~ 340 nm and a second, less pronounced, in the vicinity of ~ 265 nm. At shorter wavelengths, the absorption increases sharply to what appears to be another maximum in the region of ~ 190 nm. The apparent discontinuity amounting to ~ 20 percent in the value of *ϵ* at 275 nm is an artifact and is caused by the method used to collect the data, i.e., the wavelenth overlap between successive determinations was not sufficient to eliminate the error at that point. The difference at 275 nm is a likely indication of the maximum error due to all causes in our determination of the extinction coefficient.

Hall and Blacet indicate the absence of structure in the spectrum of N_2_O_4_. Since the error in our determination is probably of the order of 20 percent, it is uncertain whether the features observed in the region between 275–390 nm are indeed resolved structure or simply indicative of the “noise” in the experiment. In any case, no obvious regular pattern is apparent. It is important to note the large value of the extinction coefficient at short wavelengths, which indicates that extreme care is required to account adequately for N_2_O_4_ absorption in any measurement of the NO_2_ spectrum.

Although the general shape of the absorption curve agrees well with that of Hall and Blacet, the differences between the two measurements are not constant over the complete wavelength region. Between 260 and 335 nm, the two sets of measurements lie within 10 percent of each other but outside of these limits (i.e., 240–260 and 335–390 nm) the difference is closer to 20 percent. The explanation for this discrepancy may lie in the fact that in our experiments, the ratio of N_2_O_4_/NO_2_ was usually close to 4 while those of Hall reached a maximum of 1.3. Thus, the correction due to NO_2_ absorption is larger in Hall’s work than in ours.

### 3.2. NO_2_

**Figure 5:**
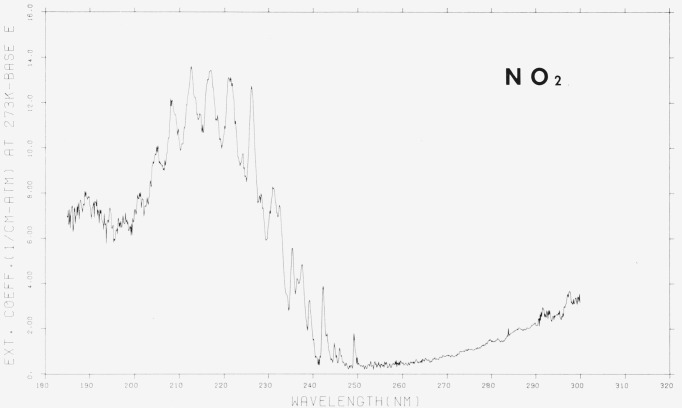
The extinction coefficients of *NO*_2_, 180–300 nm, ϵ in cm^−1^ (atm at 273 K) ^−1^ base e. [Plot is a computer reconstruction of averaged data. See text.]

**Figure 6:**
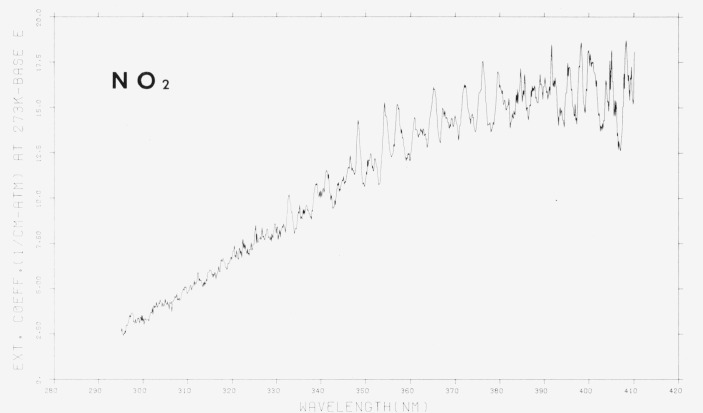
The extinction coefficients of *NO*_2_, 290–410 nm, ϵ in cm^−1^ (atm at 273 K) ^−1^ base e. [Plot is a computer reconstruction of averaged data. See text.]

The room temperature absorption spectrum of NO_2_ is shown in figures 5 and 6 and in table 3. The spectrum shown has been corrected for the contribution of N_2_O_4_ to the measured pressure. The N_2_O_4_ mole fraction was obtained from the calculated value of the equilibrium constant, *K_p_*, based upon spectroscopic and thermodynamic considerations [1]. At the pressures used, usually less than about 0.1 torr, the correction due to N_2_O_4_ concentration was less than about 0.1 percent. Similarly, a correction for the absorption due to N_2_O_4_ could be made using the measured values for *ϵ*(N_2_O_4_). In particular, at shorter wavelengths where N_2_O_4_ exhibits a very large absorption, the correction assumes more significant proportions. The experimental conditions are summarized in table 2.

The appearance of the spectrum and positions of the absorption peaks agree well with the data in the published literature [2, 3, 5]. The major difference between the present and previously published work is related to the spectral band-pass used. The larger amount of structure evident in figures 5 and 6 as compared to previous work is a consequence of the greater resolving power in the present experiments.

Bayes [9] has carefully reexamined the data of Hall and Blacet [2] and presented the values in tabular form at 0.5 nm intervals. Over the range from 250 to 410 nm our values for the extinction coefficients are 10–20 percent lower than those of Hall and Blacet. Comparison with Johnston [5] indicates good agreement (within 10 percent) over the range from 245–410 nm. Although the present values are slightly lower than those of Johnston, the latter are also lower than those of Hall and Blacet. At shorter wavelengths (245–190 nm) the agreement between Johnston and us is not quite so good, hut generally it is within 15 percent. Presumably the discrepancy may be attributed to the methods used to correct for the N_2_O_4_ absorption.

**Figure 7:**
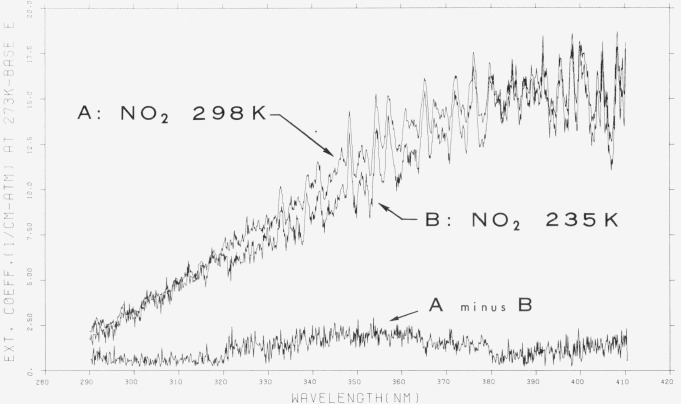
Effect of temperature upon ϵ(*NO*_2_), 290–410 nm, ϵ in cm^−1^ (atm at 273 K) ^−1^ base e. [Plot is a computer reconstruction of averaged data. See text.]

A comparison between the room-temperature and low temperature (235 K) absorption spectrum is shown in figure 7. It is clear that the discernible effect is no greater than about 10 percent and appears between 320–380 nm. It is apparent that no single feature is removed at low temperature but rather a reduction in the underlying continuum is noticed.

It may be argued that the difference spectrum is an artifact due to the incorrect numerical adjustment of the spectrum caused by the presence of N_2_O_4_ at low temperature. However, although N_2_O_4_ does have a broad absorption peak in this region (figure 4) the appearance of a “temperature effect” at 360 nm and no observable effect at ~ 290 nm where the N_2_O_4_ absorption is similar to that at 310 or 360 nm would rule out this interpretation.

We have attempted to estimate the possible sources of error in our measurements. The wavelength scale of the monochromator has been calibrated with the known emission lines of Hg and is accurate to about 0.02 nm. Inaccuracies in the pressure measurements were of the order of 1 percent and were limited to the accuracy in reading the analog output from the manometer. Significantly larger errors may result from the intensity measurement and in particular, the ratio of *I*/*I*_0_ although each individual measurement is probably accurate to within 5 percent of the “true” value. Errors in the N_2_O_4_ measurement may be more significant. For example, at a pressure of ~ 30 torr at a temperature of ~ 250 K, a one degree temperature error results in a 1.25-percent error in the N_2_O_4_ mole fraction. The method used to determine the concentration in the absorption cell involves the temperature of the cell so that an error in temperature is manifested in several ways and results in an overall N_2_O_4_ concentration which is only accurate to ± 5 percent. In all experiments, scatter between runs amounted to 10 percent or less. Consideration of those factors suggest the final value for *ϵ* (N_2_O_4_) is probably correct to within ± 20 percent, and for *ϵ* (NO_2_) to within ± 10 percent.

Table 3 lists the extinction coefficients of NO_2_ and N_2_O_4_ corrected to the equivalent pressure at 273 K.

## Figures and Tables

**Table 1:** Experimental conditions for measurement of *N*_2_*O*_4_ absorption

Wavelength (nm)	Pathlength (nm)	Temp. (K)	Sample pressure (Torr)	Mole fraction (N_2_O_4_)	Spectrum Band (nm)
185–215	0.5	250	25(4)	0.80	0.04
185–215	.095	299	117	.38	.04
195–225	.5	252	32(2)	.79	.04
215–245	.5	253	29(2)	.78	.04
215–245	.095	299	150(2)	.42	.04
225–250	.5	251	47(2)	.84	.04
245–275	.502	299	414	.58	.04
245–275	5.01	251	31(2)	.80	.04
275–305	0.502	298	443	.60	.04
275–305	5.01	253	31(2)	.78	.04
305–335	0.502	298	500	.62	.04
305–335	5.01	253	35(2)	.80	.04
335–365	0.502	274	140	.72	.02
335–365	.502	299	405	.58	.02
335–365	5.01	253	39(2)	.80	.02
345–375	0.502	299	420	.59	.02
350–380	.502	296	405	.62	.02
350–380	5.01	274	113(2)	.70	.02
380–410	0.502	296	500	.65	.02
380–410	5.01	274	114	.70	.02

**Table 2:** Experimental conditions for *NO*_2_ measurements

Wavelength (nm)	Path length (cm)	Sample pressure (torr)	Spectral band pass (nm)	Temperature
				
185–215	618	a0.100(3)	0.050	298 K
185–215	1017	.030, .040	.040	298 K
210–245	618	.090, .160, .254	.030	298 K
210–245	1017	.040, .050	.025	298 K
235–265	618	.150, .200, .350	.025	298 K
235–265	1017	.050, .060	.025	298 K
260–290	618	.280, .380, .600	.025	298 K
260–290	1017	.200, .450	.025	298 K
290–320	618	.100, .150, .250(6)	.025	298 K
310–340	1017	.050(2)	.025	298 K
320–350	618	.070, .110(6)	.025	298 K
350–380	618	.050, .100(6)	.020	298 K
350–380	219	.100(3)	.015	298 K
380–410	618	.050(3)	.015	298 K
290–350	618	.050, .070(9)	.025	235 K
320–350	618	.050, .070(3)	.025	235 K
350–380	618	.050(6)	.015	235 K
380–410	618	.030, .050(6)	.015	235 K

a Value in parentheses indicates number of runs at specified pressure.

**Table 3:** Extinction coefficients of *NO*_2_ and *N*_2_*O*_4_, 180–410 nm at 298 K and 235 K, ϵ in cm^−1^ (atm at 273 K)^−1^ base e

Wavelength (nm)	Ext. coeff. NO_2_ 298 K	Ext. coeff. NO_2_ 235 K	Ext. coeff. N_2_O_4_
185.000	6.99		1435.12
185.125	6.90		1489.56
185.250	7.28		1446.73
185.375	6.60		1455.49
185.500	6.63		1413.05
185.625	7.31		1415.12
185.750	6.43		1471.03
185.875	7.12		1428.93
186.000	7.23		1421.68
186.125	7.43		1445.05
186.250	7.46		1441.26
186.375	6.27		1515.10
186.500	6.45		1519.66
186.625	7.28		1460.57
186.750	7.39		1473.96
186.875	6.64		1457.69
187.000	7.02		1476.38
187.125	7.05		1494.71
187.250	7.45		1493.05
187.375	6.99		1502.92
187.500	7.60		1474.22
187.625	7.30		1478.17
187.750	6.82		1472.67
187.875	7.52		1558.19
188.000	7.71		1509.11
188.125	7.68		1495.25
188.250	7.34		1496.28
188.375	7.33		1456.37
188.500	7.13		1472.65
188.625	7.48		1476.16
188.750	7.55		1454.37
188.875	7.44		1493.62
189.000	8.11		1465.15
189.125	7.83		1463.46
189.250	8.10		1461.20
189.375	7.88		1440.60
189.500	7.78		1448.38
189.625	7.70		1445.53
189.750	7.88		1459.83
189.875	7.79		1435.95
190.000	7.87		1443.90
190.125	7.43		1418.47
190.250	7.36		1438.68
190.375	7.00		1427.96
190.500	6.84		1415.87
190.625	7.36		1397.85
190.750	7.36		1417.17
190.875	7.49		1405.36
191.000	7.63		1401.97
191.125	6.89		1400.23
191.250	7.66		1385.26
191.375	7.61		1397.96
191.500	7.62		1382.16
191.625	7.41		1390.34
191.750	7.76		1383.04
191.875	6.99		1383.0]
192.000	7.28		1381.11
192.125	7.13		1362.83
192.250	6.64		1373.01
192.375	7.30		1376.83
192.500	7.08		1364.96
192.625	7.28		1374.45
192.750	6.86		1352.88
192.875	6.74		1363.21
193.000	6.73		1347.79
193.125	6.61		1350.68
193.250	6.36		1343.91
193.375	7.12		1329.56
193.500	6.60		1331.66
193.625	6.58		1342.98
193.750	6.69		1322.65
193.875	5.76		1312.04
194.000	6.65		1314.07
194.125	6.81		1318.37
194.250	6.81		1325.53
194.375	6.67		1298.82
194.500	7.21		1307.81
194.625	7.38		1289.91
194.750	7.21		1287.15
194.875	7.09		1273.11
195.000	6.51		1269.54
195.125	6.40		1259.85
195.250	6.54		1256.52
195.375	6.61		1256.33
195.500	5.80		1251.84
195.625	5.99		1232.64
195.750	5.89		1239.66
195.875	6.04		1221.63
196.000	6.36		1215.02
196.125	6.31		1218.30
196.250	6.45		1215.61
196.375	6.16		1206.04
196.500	6.87		1209.04
196.625	6.92		1199.03
196.750	6.47		1190.28
196.875	6.70		1182.31
197.000	6.46		1180.36
197.125	6.78		1166.57
197.250	6.61		1157.68
197.375	6.45		1154.93
197.500	6.63		1148.60
197.625	7.03		1139.64
197.750	6.69		1111.14
197.875	6.65		1099.47
198.000	6.62		1108.31
198.125	7.16		1083.49
198.250	6.77		1076.57
198.375	6.69		1095.03
198.500	6.86		1077.86
198.625	6.60		1105.59
198.750	6.75		1079.75
198.875	6.32		1089.15
199.000	6.35		1076.41
199.125	6.49		1074.24
199.250	6.57		1081.82
199.375	6.10		1061.40
199.500	6.69		1062.31
199.625	6.14		1049.95
199.750	6.91		1042.97
199.875	6.72		1048.94
200.000	6.72		1030.98
200.125	7.30		1011.94
200.250	7.26		1025.08
200.375	7.04		1013.74
200.500	7.19		1016.60
200.625	7.65		995.60
200.750	7.96		991.83
200.875	7.75		985.55
201.000	7.73		966.78
201.125	8.03		962.74
201.250	7.84		952.24
201.375	8.07		939.16
201.500	7.34		932.56
201.625	7.59		927.16
201.750	7.80		927.35
201.875	7.70		919.00
202.000	7.72		918.44
202.125	6.95		913.48
202.250	7.16		908.92
202.375	7.45		893.77
202.500	7.45		893.91
202.625	7.40		874.78
202.750	7.50		866.46
202.875	7.83		860.91
203.000	7.59		850.68
203.125	7.48		849.85
203.250	7.84		839.54
203.375	7.83		830.97
203.500	8.43		827.94
203.625	8.59		820.62
203.750	8.26		806.66
203.875	8.33		802.67
204.000	9.22		789.38
204.125	9.23		783.10
204.250	9.43		779.09
204.375	9.29		764.28
204.500	9.51		766.24
204.625	9.68		755.55
204.750	9.76		748.27
204.875	9.67		742.25
205.000	10.08		739.04
205.125	9.77		726.37
205.250	10.13		719.61
205.375	9.94		712.35
205.500	9.33		706.30
205.625	9.42		699.53
205.750	9.14		696.19
205.875	8.95		687.54
206.000	8.98		676.20
206.125	9.14		687.38
206.250	9.26		661.62
206.375	9.14		660.08
206.500	9.00		657.77
206.625	9.11		644.67
206.750	8.98		638.26
206.875	9.50		627.86
207.000	9.20		625.71
207.125	9.43		618.75
207.250	9.74		614.16
207.375	9.80		606.47
207.500	10.23		602.78
207.625	10.46		590.97
207.750	10.42		585.00
207.875	10.99		575.64
208.000	11.36		499.61
208.125	12.20		564.13
208.250	11.82		558.14
208.375	12.10		552.35
208.500	12.13		546.85
208.625	11.87		540.10
208.750	11.66		533.24
208.875	11.55		530.16
209.000	11.48		523.04
209.125	11.49		513.46
209.250	11.44		506.32
209.375	11.15		500.45
209.500	11.02		493.22
209.625	10.53		491.29
209.750	10.74		488.43
209.875	10.87		481.50
210.000	10.34		479.60
210.125	10.20		473.98
210.250	9.96		467.10
210.375	9.86		461.92
210.500	10.06		453.41
210.625	10.20		449.39
210.750	10.17		443.48
210.875	10.19		441.01
211.000	10.58		433.79
211.125	10.92		430.67
211.250	10.87		425.20
211.375	11.11		417.67
211.500	11.43		412.24
211.625	11.92		405.19
211.750	11.89		406.99
211.875	12.25		400.57
212.000	12.34		392.69
212.125	12.52		388.81
212.250	12.65		382.41
212.375	12.85		378.31
212.500	13.14		375.68
212.625	13.47		372.30
212.750	13.63		365.72
212.875	13.44		363.96
213.000	13.21		358.44
213.125	12.84		354.46
213.250	12.41		347.51
213.375	12.28		342.43
213.500	12.23		340.95
213.625	12.25		333.78
213.750	12.11		327.59
213.875	11.77		325.05
214.000	11.46		318.52
214.125	11.51		320.46
214.250	11.22		316.63
214.375	11.30		305.72
214.500	11.35		302.35
214.625	11.55		305.19
214.750	11.45		291.06
214.875	11.44		294.52
215.000	10.81		295.14
215.125	10.67		287.13
215.250	11.05		285.43
215.375	10.66		288.95
215.500	10.72		293.68
215.625	11.11		284.66
215.750	11.43		287.52
215.875	12.18		281.27
216.000	12.46		278.41
216.125	12.79		274.74
216.250	12.87		277.77
216.375	13.05		270.61
216.500	13.00		266.72
216.625	13.08		255.81
216.750	13.39		254.71
216.875	13.37		261.66
217.000	13.43		249.59
217.125	13.45		244.93
217.250	13.30		242.00
217.375	13.01		237.84
217.500	12.66		233.99
217.625	12.66		236.10
217.750	12.22		233.91
217.875	11.98		232.83
218.000	11.75		219.62
218.125	11.20		225.42
218.250	11.30		222.08
218.375	11.29		220.51
218.500	11.17		210.23
218.625	11.47		205.43
218.750	11.23		205.34
218.875	11.18		211.63
219.000	10.53		201.90
219.125	10.34		201.07
219.250	10.44		199.81
219.375	10.20		190.04
219.500	9.96		191.92
219.625	10.14		189.35
219.750	10.26		184.20
219.875	10.52		184.96
220.000	10.64		179.65
220.125	10.99		182.78
220.250	10.83		177.87
220.375	11.24		178.31
220.500	11.66		170.95
220.625	12.26		165.77
220.750	12.53		174.80
220.875	12.97		171.59
221.000	13.13		164.99
221.125	13.08		171.63
221.250	13.04		156.75
221.375	12.89		163.22
221.500	13.14		161.85
221.625	13.02		156.90
221.750	12.71		149.78
221.875	12.88		138.57
222.000	12.53		149.25
222.125	12.23		148.80
222.250	12.16		144.25
222.375	11.49		134.67
222.500	11.04		142.57
222.625	11.21		141.75
222.750	10.87		132.37
222.875	10.53		136.72
223.000	10.07		134.86
223.125	9.59		129.75
223.250	9.57		123.02
223.375	9.40		130.14
223.500	9.40		129.51
223.625	9.19		122.44
223.750	9.35		125.91
223.875	9.34		124.61
224.000	9.40		122.33
224.125	9.63		119.06
224.250	9.76		125.55
224.375	9.74		122.10
224.500	9.07		119.00
224.625	9.17		113.73
224.750	8.70		119.06
224.875	8.78		115.24
225.000	8.71		110.60
225.125	8.49		108.02
225.250	8.77		106.09
225.375	9.20		104.94
225.500	9.68		100.06
225.625	10.04		104.52
225.750	10.91		100.81
225.875	11.30		104.02
226.000	11.99		104.88
226.125	12.56		92.47
226.250	12.75		97.89
226.375	12.50		95.76
226.500	12.28		91.87
226.625	11.77		95.83
226.750	11.24		86.61
226.875	10.61		90.89
227.000	9.89		89.88
227.125	9.44		90.61
227.250	9.04		82.91
227.375	8.47		84.62
227.500	8.20		86.00
227.625	7.55		76.59
227.750	7.60		85.84
227.875	7.69		85.22
228.000	7.90		76.98
228.125	7.82		85.19
228.250	7.54		72.86
228.375	8.00		74.00
228.500	7.88		80.39
228.625	7.69		77.17
228.750	7.30		73.80
228.875	7.35		79.25
229.000	6.90		76.46
229.125	6.73		72.83
229.250	6.43		76.44
229.375	6.08		76.10
229.500	5.91		69.57
229.625	5.91		64.62
229.750	5.98		73.00
229.875	6.00		72.98
230.000	6.52		68.66
230.125	6.64		62.98
230.250	6.97		68.12
230.375	7.23		69.95
230.500	7.08		68.89
230.625	7.55		63.53
230.750	7.78		59.49
230.875	7.86		61.43
231.000	8.30		60.82
231.125	8.19		58.75
231.250	8.23		59.75
231.375	8.10		61.43
231.500	7.88		59.29
231.625	7.57		60.42
231.750	7.54		58.54
231.875	7.12		57.57
232.000	7.14		56.50
232.125	6.93		54.46
232.250	6.98		56.54
232.375	7.16		58.55
232.500	7.48		60.39
232.625	7.43		54.39
232.750	7.25		57.91
232.875	6.93		54.77
233.000	6.42		50.30
233.125	6.11		56.00
233.250	5.67		49.04
233.375	5.12		54.58
233.500	4.69		50.31
233.625	4.29		51.51
233.750	3.85		55.63
233.875	3.67		48.91
234.000	3.55		53.60
234.125	3.48		55.17
234.250	3.47		46.90
234.375	3.38		49.60
234.500	3.09		50.23
234.625	2.78		49.48
234.750	2.89		50.28
234.875	3.38		47.36
235.000	3.98		47.37
235.125	4. 9		45.90
235.250	4.94		49.00
235.375	5.59		47.79
235.500	5.54		49.24
235.625	5.19		54.10
235.750	4.47		44.08
235.875	4.19		46.98
236.000	3.78		38.78
236.125	3.51		48.57
236.250	3.71		49.89
236.375	3.93		44.55
236.500	4.24		47.09
236.625	4.19		49.03
236.750	4.10		48.72
236.875	3.98		44.72
237.000	4.01		47.77
237.125	4.15		36.85
237.250	4.28		48.97
237.375	4.59		38.62
237.500	4.80		37.57
237.625	4.87		30.41
237.750	4.60		39.40
237.875	4.26		33.52
238.000	3.87		47.94
238.125	3.42		49.02
238.250	3.09		32.33
238.375	2.85		43.17
238.500	2.45		37.64
238.625	2.37		47.79
238.750	2.08		39.67
238.875	1.90		41.41
239.000	2.26		38.66
239.125	3.06		38.97
239.250	3.26		40.21
239.375	3.25		37.98
239.500	2.84		36.42
239.625	2.61		43.61
239.750	2.36		47.05
239.875	2.01		36.11
240.000	1.80		41.15
240.125	1.48		34.54
240.250	1.55		31.80
240.375	1.27		38.66
240.500	1.00		37.36
240.625	0.70		38.91
240.750	.81		41.61
240.875	.72		35.66
241.000	.71		31.30
241.125	.52		31.71
241.250	0.38		39.45
241.375	0.68		29.13
241.500	0.37		34.54
241.625	0.80		39.27
241.750	0.71		41.75
241.875	1.09		37.20
242.000	2.00		30.89
242.125	3.14		38.31
242.250	3.80		29.58
242.375	3.90		35.10
242.500	3.23		36.23
242.625	2.85		39.33
242.750	2.24		36.16
242.875	1.82		32.52
243.000	1.66		32.31
243.125	1.69		30.95
243.250	1.80		35.41
243.375	1.45		33.41
243.500	1.33		40.46
243.625	1.03		32.10
243.750	1.00		38.57
243.875	0.85		39.85
244.000	.69		36.30
244.125	.59		33.61
244.250	.69		32.40
244.375	.63		34.62
244.500	.48		34.25
244.625	.65		32.48
244.750	1.03		37.99
244.875	1.37		33.69
245.000	1.17		31.13
245.125	1.02		35.69
245.250	0.71		27.16
245.375	.84		26.32
245.500	.59		30.89
245.625	.46		27.72
245.750	.55		27.15
245.875	.70		27.21
246.000	1.17		26.99
246.125	1.09		25.54
246.250	0.88		27.48
246.375	.77		25.67
246.500	.82		25.90
246.625	.59		24.13
246.750	.50		26.35
246.875	.54		29.55
247.000	.49		23.02
247.125	.43		27.13
247.250	.34		28.88
247.375	.40		26.56
247.500	0.50		29.13
247.625	.39		29.85
247.750	.32		26.45
247.875	.26		28.03
248.000	.21		29.57
248.125	.27		28.83
248.250	.39		27.03
248.375	.27		30.55
248.500	.39		27.08
248.625	.38		27.21
248.750	.39		29.19
248.875	.23		29.13
249.000	.37		31.09
249.125	1.05		29.11
249.250	1.81		26.61
249.375	1.52		25.46
249.500	1.09		25.22
249.625	.94		27.21
249.750	.80		27.91
249.875	.44		28.75
250.000	.76		28.27
250.125	.38		27.43
250.250	.50		26.99
250.375	.44		27.05
250.500	.40		26.52
250.625	.38		24.83
250.750	.33		25.06
250.875	.38		31.50
251.000	.37		26.77
251.125	.31		21.71
251.250	.34		24.06
251.375	.40		31.14
251.500	.30		27.51
251.625	.19		17.90
251.750	.35		27.87
251.875	.37		26.29
252.000	.24		26.27
252.125	.23		26.16
252.250	.33		27.98
252.375	.42		25.36
252.500	.42		26.20
252.625	.44		29.44
252.750	.33		26.10
252.875	.47		25.12
253.000	.59		25.42
253.125	.32		25.64
253.250	.36		24.76
253.375	.39		25.62
253.500	.38		24.86
253.625	.18		25.98
253.750	.39		24.78
253.875	.54		25.80
254.000	.56		24.06
254.125	.40		26.61
254.250	.47		25.59
254.375	.28		25.23
254.500	.39		25.51
254.625	.42		26.08
254.750	.56		24.79
254.875	.35		27.30
255.000	.39		26.45
255.125	.24		24.75
255.250	.45		25.10
255.375	.40		25.00
255.500	.46		25.41
255.625	.32		24.07
255.750	.38		24.56
255.875	.41		24.66
256.000	.29		24.48
256.125	.56		23.86
256.250	.47		24.02
256.375	.40		24.53
256.500	.55		24.53
256.625	.42		25.04
256.750	.26		24.64
256.875	.29		24.81
257.000	.36		24.31
257.125	.35		24.29
257.250	.48		23.92
257.375	.38		24.40
257.500	.27		24.23
257.625	* .31		24.22
257.750	.41		24.32
257.875	.46		24.59
258.000	.44		24.21
258.125	.48		24.42
258.250	.42		24.65
258.375	.40		24.79
258.500	.58		24.11
258.625	.42		24.34
258.750	.63		23.69
258.875	.30		24.23
259.000	.51		24.58
259.125	.47		24.19
259.250	.38		24.00
259.375	.56		24.52
259.500	.37		23.90
259.625	.61		23.88
259.750	.55		23.98
259.875	.47		24.28
260.000	0.51		23.93
260.125	.46		24.42
260.250	.51		23.98
260.375	.38		24.37
260.500	.41		23.86
260.625	.43		24.12
260.750	.46		24.17
260.875	.52		24.10
261.000	.44		24.28
261.125	.41		23.84
261.250	.38		24.02
261.375	.41		23.91
261.500	.53		24.13
261.625	.42		23.37
261.750	.39		23.86
261.875	.54		23.90
262.000	.55		24.15
262.125	.48		23.64
262.250	.53		23.60
262.375	.54		23.71
262.500	.45		23.56
262.625	.57		23.57
262.750	.45		23.80
262.875	.54		23.95
263.000	.60		23.63
263.125	.65		23.85
263.250	.63		23.53
263.375	.55		24.09
263.500	.50		23.91
263.625	.55		23.83
263.750	.47		23.32
263.875	.52		23.32
264.000	.56		23.12
264.125	.57		22.96
264.250	.49		22.74
264.375	.64		23.00
264.500	.49		29.56
264.625	.69		22.66
264.750	.61		22.97
264.875	.63		23.15
265.000	.55		23.17
265.125	.66		23.22
265.250	.72		23.03
265.375	.66		23.57
265.500	.67		23.00
265.625	.60		23.08
265.750	.63		22.87
265.875	.61		22.79
266.000	.64		22.71
266.125	.58		22.82
266.250	0.50		23.02
266.375	.56		22.77
266.500	.60		23.23
266.625	.53		22.96
266.750	.59		22.98
266.875	.69		22.58
256.000	.65		22.57
267.125	.68		22.82
267.250	.67		21.90
267.375	.75		22.22
267.500	.65		22.42
267.625	.64		22.00
267.750	.68		22.30
267.875	.67		22.31
268.000	.64		22.79
268.125	.63		22.32
268.250	.71		22.18
268.375	.75		22.16
268.500	.75		21.57
268.625	.75		21.98
268.750	.77		21.63
268.875	.72		21.87
269.000	.75		21.67
269.125	.84		21.68
269.250	.81		21.60
269.375	.80		21.65
269.500	.77		21.02
269.625	.80		21.26
269.750	.78		21.11
269.875	.82		22.32
270.000	.84		21.25
270.125	.84		20.97
270.250	.80		20.92
270.375	.85		20.35
270.500	.80		21.48
270.625	.82		20.83
270.750	.80		20.45
270.875	.73		20.72
271.000	.82		21.23
271.125	.85		20.90
271.250	.84		20.63
271.375	.76		20.60
271.500	.76		20.74
271.625	.78		20.73
271.750	.78		14.99
271.875	.81		20.03
272.000	.83		20.23
272.125	.85		20.82
272.250	.79		20.67
272.375	.92		20.43
272.500	.86		20.48
272.625	.89		20.76
272.750	.89		20.63
272.875	1.00		20.17
273.000	1.02		20.08
273.125	.99		20.34
273.250	.98		20.08
273.375	1.02		20.09
273.500	1.00		19.69
273.625	.98		19.69
273.750	1.02		19.66
273.875	1.05		19.49
274.000	.98		19.82
274.125	1.05		19.96
274.250	1.08		19.38
274.375	1.08		19.83
274.500	1.03		19.25
274.625	1.08		16.36
274.750	1.03		19.73
274.875	1.13		19.72
275.000	1.08		16.89
275.125	1.09		16.43
275.250	1.09		16.63
275.375	1.08		16.75
275.500	1.11		16.64
275.625	1.10		16.29
275.750	1.07		16.09
275.875	1.06		16.24
276.000	1.06		16.70
276.125	1.07		16.00
276.250	1.11		16.08
276.375	1.10		15.83
276.500	1.12		15.82
276.625	1.14		15.92
276.750	1.10		15.93
276.875	1.19		15.84
277.000	1.14		15.95
277.125	1.15		15.57
277.250	1.13		15.12
277.375	1.19		15.57
277.500	1.15		15.56
277.625	1.15		15.20
277.750	1.23		15.45
277.875	1.20		15.93
278.000	1.29		15.82
278.125	1.26		15.32
278.250	1.31		15.67
278.375	1.24		15.92
278.500	1.31		15.52
278.625	1.29		15.50
278.750	1.28		15.46
278.875	1.40		15.31
279.000	1.35		15.30
279.125	1.32		15.24
279.250	1.41		15.22
279.375	1.48		14.44
279.500	1.50		14.97
279.625	1.47		14.97
279.750	1.55		14.76
279-875	1.48		14.61
280.000	1.49		14.66
280.125	1.41		14.63
280.250	1.49		14.51
280.375	1.42		14.42
280.500	1.43		14.32
280.625	1.47		14.25
280.750	1.42		14.46
280.875	1.44		14.07
281.000	1.50		14.23
281.125	1.48		13.76
281.250	1.52		14.58
281.375	1.52		14.02
281.500	1.59		13.97
281.625	1.55		14.15
281.750	1.52		14.02
281.875	1.47		14.07
282.000	1.44		13.75
282.125	1.41		13.61
282.250	1.49		13.86
282.375	1.47		13.84
282.500	1.43		13.39
282.625	1.43		13.83
282.750	1.49		13.93
282.875	1.45		13.95
283.000	1.50		13.64
283.125	1.54		13.62
283.250	1.60		13.89
283.375	1.60		15.94
283.500	1.65		13.11
283.625	1.72		13.15
283.750	1.67		13.30
283.875	2.04		13.61
284.000	1.68		13.26
284.125	1.77		13.25
284.250	1.76		13.10
284.375	1.81		13.21
284.500	1.82		12.93
284.625	1.83		12.50
284.750	1.85		13.00
284.875	1.88		12.48
285.000	1.88		12.14
285.125	1.86		12.37
285.250	1.94		12.16
285.375	1.91		13.22
285.500	1.95		12.38
285.625	1.97		12.63
285.750	1.95		12.34
285.875	2.01		12.12
286.000	1.96		11.89
286.125	2.05		12.10
286.250	2.05		12.02
286.375	2.06		12.05
286.500	2.02		11.94
286.625	1.98		11.60
286.750	2.00		12.10
286.875	1.97		11.71
287.000	1.98		11.93
287.125	1.93		11.79
287.250	1.93		11.67
287.375	2.01		11.48
287.500	1.98		11.44
287.625	1.98		11.77
287.750	1.95		11.29
287.875	2.00		11.25
288.000	2.06		11.33
288.125	2.05		11.05
288.250	2.01		11.06
288.375	2.02		11.52
288.500	2.03		11.30
288.625	2.04		11.09
288.750	2.07		10.95
288.875	2.10		11.43
289.000	2.12		10.93
289.125	2.18		11.31
289.250	2.16		11.38
289.375	2.18		11.42
289.500	2.23		10.88
289.625	2.22		10.71
289.750	2.21		11.04
289.875	2.30		10.93
290.000	2.20	1.82	10.62
290.125	2.13	1.70	10.70
290.250	2.17	1.72	10.74
290.375	2.17	2.04	10.40
290.500	2.15	1.60	10.56
290.625	2.45	1.60	10.59
290.750	2.48	1.39	10.52
290.875	2.10	2.02	10.38
291.000	2.66	2.01	10.59
291.125	2.37	2.01	10.20
291.250	2.79	2.01	10.15
291.375	2.53	1.72	10.07
291.500	2.83	1.91	10.32
291.625	2.97	2.64	9.86
291.750	2.85	2.06	10.17
291.875	2.82	1.94	9.90
292.000	2.52	2.16	10.23
292.125	2.69	1.56	9.87
292.250	2.52	2.04	9.73
292.375	2.50	2.62	10.48
292.500	2.84	2.36	9.70
292.625	2.69	2.33	10.26
292.750	2.43	2.17	10.07
292.875	2.84	2.27	10.01
293.000	2.62	1.96	9.80
293.125	2.85	2.30	9.68
293.250	2.63	2.27	9.66
293.375	2.49	1.84	9.52
293.500	2.34	2.16	9.80
293.625	2.33	1.50	9.42
293.750	2.46	2.20	9.61
293.875	2.59	2.50	9.21
294.000	2.55	2.36	9.27
294.125	2.38	2.02	9.43
294.250	2.62	1.76	9.56
294.375	2.50	2.54	9.28
294.500	2.60	1.86	10.03
294.625	2.61	2.43	9.69
294.750	2.65	2.69	8.96
294.875	2.62	2.66	9.11
295.000	2.60	2.29	9.10
295.125	2.72	2.51	9.17
295.250	2.63	2.01	9.04
295.375	2.84	1.64	9.39
295.500	2.50	2.29	8.94
295.625	2.46	2.31	8.65
295.750	2.39	1.92	8.82
295.875	2.58	2.28	9.21
296.000	2.50	2.76	8.77
296.125	2.70	2.20	8.75
296.250	2.53	2.56	8.45
296.375	2.98	2.53	8.74
296.500	3.00	2.40	8.50
296.625	2.94	2.77	8.94
296.750	3.08	2.52	9.42
296.875	3.12	2.45	8.70
297.000	3.27	2.98	8.47
297.125	3.44	2.89	8.71
297.250	3.55	3.24	8.64
297.375	3.39	3.15	8.59
297.500	3.62	3.13	8.52
297.625	3.67	2.89	8.64
297.750	3.65	3.15	8.72
297.875	3.59	3.86	8.35
298.000	3.15	3.10	8.56
298.125	3.21	2.96	8.48
298.250	3.08	2.83	8.55
298.375	3.10	2.60	8.84
298.500	3.34	3.07	8.66
298.625	3.09	3.37	8.42
298.750	3.12	2.72	8.81
298.875	3.26	2.96	8.35
299.000	3.38	3.74	8.35
299.125	3.39	3.01	7.91
299.250	3.19	2.78	8.01
299.375	3.07	3.01	8.02
299.500	3.43	3.34	8.37
299.625	3.40	3.18	8.27
299.750	3.20	2.84	8.48
299.875	3.56	2.85	7.82
300.000	3.15	2.93	8.32
300.125	3.37	2.98	8.51
300.250	3.01	2.37	8.37
300.375	3.17	2.99	8.39
300.500	3.38	2.86	8.29
300.625	3.27	3.32	7.99
300.750	3.25	3.03	8.45
300.875	3.29	2.96	8.02
301.000	3.30	3.27	8.21
301.125	3.28	3.25	8.41
301.250	3.18	2.72	8.00
301.375	3.49	2.99	7.93
301.500	3.50	2.71	8.23
301.625	3.65	3.05	7.84
301.750	3.71	3.87	8.20
301.875	3.64	3.38	8.21
302.000	3.73	3.51	8.30
302.125	3.98	3.41	8.45
302.250	3.55	3.54	8.56
302.375	3.86	3.25	8.05
302.500	4.10	3.70	8.15
302.625	3.93	3.84	7.97
302.750	4.03	3.42	8.09
302.875	3.97	4.19	7.68
303.000	4.28	4.50	7.86
303.125	4.11	3.73	8.14
303.250	3.93	3.85	8.35
303.375	4.06	3.96	8.45
303.500	4.13	3.65	8.26
303.625	4.23	4.11	8.07
303.750	4.15	4.34	8.08
303.875	4.42	4.12	7.61
304.000	4.29	3.90	7.57
304.125	4.23	3.95	8.37
304.250	4.01	3.91	8.43
304.375	4.02	4.15	8.45
304.500	4.24	3.79	8.10
304.625	4.02	4.01	8.26
304.750	4.04	3.50	8.23
304.875	4.13	3.94	8.38
305.000	4.45	4.48	8.18
305.125	4.20	3.69	7.75
305.250	4.17	3.38	7.60
305.375	4.08	3.53	7.86
305.500	4.32	3.81	7.55
305.625	4.09	3.82	7.73
305.750	4.11	4.11	7.05
305.875	3.83	3.69	7.51
306.000	4.25	4.02	7.35
306.125	4.19	3.13	8.07
306.250	4.17	3.69	8.40
306.375	4.32	4.10	8.08
306.500	3.69	3.88	6.92
306.625	3.98	3.60	8.17
306.750	4.31	3.74	7.98
306.875	4.32	4.21	8.07
307.000	4.39	3.96	7.43
307.125	4.46	4.50	7.26
307.250	4.61	4.56	8.12
307.375	4.48	5.19	7.92
307.500	4.53	4.23	7.68
307.625	4.73	4.40	7.78
307.750	4.35	3.78	7.98
307.875	4.45	4.38	7.65
308.000	4.35	4.07	7.87
308.125	4.51	3.63	8.40
308.250	4.33	4.44	7.59
308.375	4.39	3.85	8.17
308.500	4.42	3.90	7.40
308.625	4.67	4.29	7.66
308.750	4.78	4.42	8.42
308.875	4.86	4.93	7.96
309.000	4.94	4.99	7.76
309.125	5.01	4.35	7.78
309.250	4.88	4.87	7.71
309.375	5.12	5.17	8.05
309.500	5.03	4.59	8.02
309.625	5.12	5.00	8.11
309.750	5.06	4.76	8.12
309.875	4.78	4.83	7.95
310.000	4.72	4.91	8.20
310.125	4.78	4.37	8.39
310.250	4.91	4.41	8.51
310.375	4.90	5.06	8.00
310.500	4.97	4.60	8.20
310.625	5.18	4.41	8.65
310.750	5.19	5.06	8.22
310.875	4.90	5.02	8.91
311.000	5.05	4.56	8.31
311.125	5.15	4.64	8.01
311.250	5.15	4.82	7.97
311.375	5.24	4.85	8.81
311.500	5.47	4.85	8.60
311.625	5.15	4.73	8.45
311.750	5.34	4.69	8.40
311.875	5.32	4.77	8.58
312.000	5.27	5.75	8.49
312.125	5.40	5.17	8.43
312.250	5.61	6.10	8.08
312.375	5.92	5.29	8.81
312.500	5.80	5.03	8.61
312.625	5.40	4.77	9.07
312.750	5.56	5.25	9.41
312.875	5.42	5.19	9.44
313.000	5.47	5.15	9.26
313.125	5.45	5.15	9.79
313.250	5.28	4.88	9.21
313.375	5.15	4.89	9.67
313.500	5.08	5.04	9.08
313.625	5.37	4.94	8.85
313.750	5.13	4.92	9.16
313.875	5.42	5.31	9.42
314.000	5.22	4.96	9.68
314.125	5.30	5.13	9.81
314.250	5.52	5.03	9.64
314.375	5.41	5.43	9.74
314.500	5.88	5.01	9.90
314.625	5.91	5.14	10.31
314.750	5.92	5.36	9.82
314.875	5.95	5.49	9.78
315.000	6.05	5.89	9.74
315.125	6.01	5.20	10.01
315.250	6.02	5.85	10.04
315.375	5.90	5.74	9.90
315.500	5.77	5.96	10.93
315.625	5.82	5.29	10.44
315.750	5.60	4.97	10.81
315.875	5.81	5.26	11.01
316.000	5.73	5.04	11.03
316.125	5.92	4.60	10.79
316.250	5.84	4.95	10.67
316.375	5.50	5.26	10.75
316.500	5.80	5.08	10.61
316.625	5.78	5.45	10.84
316.750	5.93	5.54	10.65
316.875	6.03	5.74	11.04
317.000	6.26	6.00	10.98
317.125	5.91	6.39	10.85
317.250	5.92	5.94	10.76
317.375	6.32	6.28	11.25
317.500	6.63	6.83	11.10
317.625	6.56	6.18	10.81
317.750	6.48	6.36	11.20
317.875	6.64	6.15	11.28
318.000	6.67	6.47	11.43
318.125	6.52	6.04	11.83
318.250	6.46	5.93	11.54
318.375	6.40	6.23	12.15
318.500	6.17	6.13	12.07
318.625^	6.18	6.02	12.00
318.750	6.15	5.82	11.86
318.875	6.04	5.81	12.11
319.000	6.22	5.85	12.01
319.125	6.42	6.49	12.70
319.250	6.38	5.77	12.30
319.375	6.41	5.74	12.65
319.500	6.55	6.23	11.87
319.625	6.70	6.51	12.51
319.750	6.77	6.34	12.34
319.875	6.57	6.40	12.21
320.000	6.82	6.31	12.20
320.125	7.08	6.50	12.76
320.250	7.06	5.98	12.49
320.375	7.16	6.44	12.76
320.500	7.41	6.06	12.81
320.625	6.75	6.32	12.43
320.750	6.86	6.29	12.52
320.875	6.66	6.35	12.67
321.000	7.13	5.52	13.40
321.125	6.91	5.15	13.21
321.250	6.77	5.51	13.10
321.375	6.59	5.67	13.13
321.500	6.70	5.41	13.36
321.625	7.26	6.16	13.72
321.750	7.18	4.92	13.73
321.875	6.87	6.35	13.01
322.000	7.12	5.94	13.74
322.125	6.98	5.91	13.55
322.250	6.69	5.73	13.99
322.375	7.48	6.01	14.37
322.500	7.76	6.22	13.52
322.625	7.21	5.94	14.37
322.750	7.54	6.40	14.11
322.875	7.17	5.84	14.80
323.000	7.44	6.56	14.73
323.125	7.11	6.09	14.70
323.250	7.03	6.34	13.87
323.375	6.91	6.03	14.69
323.500	7.24	6.12	14.68
323.625	6.89	6.37	14.29
323.750	6.98	6.12	14.57
323.875	6.80	5.86	14.74
324.000	7.19	6.02	14.64
324.125	7.15	5.97	14.93
324.250	7.34	5.98	14.92
324.375	7.57	5.93	15.12
324.500	7.09	6.06	15.09
324.625	7.19	6.54	15.30
324.750	7.45	6.42	15.29
324.875	7.46	5.95	15.41
325.000	7.49	6.83	14.69
325.125	8.04	6.65	15.64
325.250	8.26	6.55	15.62
325.375	8.53	6.94	15.17
325.000	7.48	6.78	15.88
325.625	8.05	7.24	15.84
325.750	7.55	6.61	15.28
325.875	7.56	6.30	15.66
326.000	7.74	5.86	15.42
326.125	7.79	6.41	15.58
326.250	7.77	6.34	15.90
326.375	7.89	6.71	16.15
326.500	7.68	7.16	16.27
326.625	7.83	6.86	16.19
326.750	8.42	7.21	16.55
326.875	7.95	5.93	16.85
327.000	7.82	6.32	16.12
327.125	7.93	6.63	16.39
327.250	7.73	6.47	16.32
327.375	7.68	6.42	16.40
327.500	8.00	6.77	16.53
327.625	7.95	6.77	16.76
327.750	8.17	6.97	16.55
327.875	8.32	6.96	17.02
328.000	8.27	7.03	16.79
328.125	7.80	6.60	17.16
328.250	7.87	7.03	17.01
328.375	7.85	6.91	16.90
328.500	7.72	6.65	17.06
328.625	7.68	6.81	16.58
328.750	7.90	6.14	16.77
328.875	7.58	6.36	17.23
329.000	8.06	7.51	16.96
329.125	7.86	7.15	17.62
329.250	7.78	7.62	17.42
329.375	8.10	7.32	19.84
329.500	8.26	7.44	18.03
329.625	8.50	7.12	18.14
329.750	8.63	7.63	18.13
329.875	8.43	7.92	17.65
330.000	8.03	7.82	17.69
330.125	8.36	6.80	17.84
330.250	8.45	7.07	18.05
330.375	7.66	6.86	18.07
330.500	7.93	7.49	17.72
330.625	8.01	7.08	17.60
330.750	8.28	7.02	18.63
330.875	7.82	6.87	18.44
331.000	8.20	6.59	18.24
331.125	8.28	6.71	18.17
331.250	8.37	7.38	18.49
331.375	8.50	7.32	17.78
331.500	8.33	6.74	18.95
331.625	8.60	6.84	18.44
331.750	8.43	6.95	18.24
331.875	8.39	6.68	18.62
332.000	8.08	7.10	18.29
332.125	8.44	7.12	18.71
332.250	8.50	7.86	19.26
332.375	8.99	7.76	18.98
332.500	9.39	8.38	18.52
332.625	9.94	7.84	21.33
332.750	10.05	8.80	18.52
332.875	10.20	8.83	18.51
333.000	10.02	8.76	19.72
333.125	9.77	9.10	19.97
333.250	9.70	8.70	19.45
333.375	9.21	8.83	19.00
333.500	9.23	7.64	19.20
333.625	8.83	7.80	18.70
333.750	8.69	7.38	19.17
333.875	8.46	7.57	19.80
334.000	8.01	6.98	19.54
334.125	8.14	6.60	19.83
334.250	8.35	7.20	19.48
334.375	8.33	7.51	19.42
334.500	8.29	7.05	20.05
334.625	8.56	7.56	19.20
334.750	9.00	7.62	19.44
334.875	9.11	8.74	19.74
335.000	9.28	8.43	19.41
335.125	9.12	7.82	21.11
335.250	9.67	7.89	19.94
335.375	9.17	7.83	22.06
335.500	8.83	7.66	20.92
335.625	9.01	7.79	20.62
335.750	8.85	8.03	20.97
335.875	8.89	7.15	22.42
336.000	9.43	7.86	20.71
336.125	9.09	6.92	22.15
336.250	9.40	7.49	21.95
336.375	9.06	8.12	23.00
336.500	9.32	8.59	21.15
336.625	9.53	8.33	21.60
336.750	9.60	8.43	21.34
336.875	9.62	7.53	22.54
337.000	9.31	7.17	22.03
337.125	9.37	8.14	21.91
337.250	9.24	7.33	21.89
337.375	9.13	7.64	22.30
337.500	9.12	7.24	21.85
337.625	8.93	7.83	21.85
337.750	8.98	6.90	22.51
337.875	8.81	7.16	22.68
338.000	9.35	7.10	22.03
338.125	9.27	7.54	22.05
338.250	9.69	8.11	21.86
338.375	9.89	8.58	22.30
338.500	10.34	9.12	22.43
338.625	10.48	9.29	22.33
338.750	10.70	9.85	22.27
338.875	10.80	9.64	22.14
339.000	10.72	9.45	22.15
339.125	10.85	9.19	22.16
339.250	10.55	9.12	22.57
339.375	10.22	9.04	22.53
339.500	10.08	8.32	23.61
339.625	10.10	8.52	23.11
339.750	10.37	8.04	22.01
339.875	9.99	8.67	23.01
340.000	10.43	8.69	22.27
340.125	10.30	8.82	22.55
340.250	10.04	8.33	21.67
340.375	10.35	8.63	22.49
340.500	10.53	8.33	21.93
340.625	10.55	8.55	22.20
340.750	10.28	9.10	21.55
340.875	10.55	8.81	22.61
341.000	11.20	9.30	22.36
341.125	11.41	9.68	22.28
341.250	11.55	9.31	21.98
341.375	11.48	9.68	22.34
341.500	11.37	9.86	22.68
341.625	11.03	9.92	22.02
341.750	11.37	9.92	21.98
341.875	10.62	9.52	21.87
342.000	10.30	8.92	21.98
342.125	10.19	8.55	22.16
342.250	9.85	8.85	22.09
342.375	10.20	7.89	21.61
342.500	9.94	7.92	21.88
342.625	9.46	8.36	22.28
342.750	9.42	7.56	21.90
342.875	9.52	8.22	21.87
343.000	9.53	8.22	22.29
343.125	9.90	8.38	22.36
343.250	9.62	8.37	21.97
343.375	9.99	8.88	21.88
343.500	10.19	9.06	22.37
343.625	10.54	8.55	21.41
343.750	10.82	9.45	21.37
343.875	10.69	8.53	22.45
344.000	10.91	8.79	22.08
344.125	10.33	8.61	21.51
344.250	10.52	8.84	21.78
344.375	10.41	8.87	21.09
344.500	10.64	8.58	22.41
344.625	10.82	8.74	23.66
344.750	10.93	8.46	24.97
344.875	11.03	8.68	21.63
345.000	10.94	9.21	20.99
345.125	11.22	9.25	21.71
345.250	10.92	9.54	21.27
345.375	10.74	8.87	21.47
345.500	10.95	9.01	21.50
345.625	10.86	9.05	21.34
345.750	11.11	9.72	21.43
345.875	11.30	9.41	21.10
346.000	11.54	9.57	21.28
346.126	11.67	9.36	21.63
346.250	11.72	9.74	20.79
346.375	11.61	9.93	20.78
346.500	12.06	10.05	20.14
346.625	12.36	10.10	20.80
346.750	11.99	10.22	20.43
346.875	11.83	10.32	20.36
347.000	11.50	9.94	20.12
347.125	11.68	9.80	20.35
347.250	11.48	9.57	20.14
347.375	11.53	9.48	19.81
347.500	11.29	9.71	19.93
347.625	11.32	9.50	20.43
347.750	11.59	9.89	19.36
347.875	11.82	10.09	19.93
348.000	12.96	10.69	19.36
348.125	13.46	11.51	19.25
348.250	13.84	12.39	19.51
348.375	14.33	13.15	18.33
348.500	14.05	12.55	18.73
348.625	13.68	12.14	18.94
348.750	13.15	11.77	19.32
348.875	12.56	11.21	19.27
349.000	12.40	10.40	18.85
349.125	11.84	10.46	19.15
349.250	11.22	9.31	18.96
349.375	10.78	8.94	18.84
349.500	10.75	9.70	18.24
349.625	10.88	8.72	18.79
349.750	10.68	8.64	19.04
349.875	10.63	9.18	19.80
350.000	11.02	8.37	19.75
350.125	11.13	9.52	17.34
350.250	11.46	9.67	17.16
350.375	11.29	10.24	16.88
350.500	12.13	10.32	17.08
350.625	11.72	10.55	16.84
350.750	12.14	10.27	16.74
350.875	12.05	10.17	16.79
351.000	12.15	10.84	16.91
351.125	12.46	11.00	16.36
351.250	12.44	10.50	17.09
351.375	12.04	10.76	16.45
351.500	11.63	10.56	16.90
351.625	11.80	9.91	16.57
351.750	11.46	9.88	16.90
351.875	11.53	9.60	16.08
352.000	11.93	10.10	16.18
352.125	12.21	10.91	16.68
352.250	12.14	10.15	15.79
352.375	11.92	10.25	16.35
352.500	11.78	9.94	16.04
352.625	11.66	9.90	15.78
352.750	11.31	9.20	15.67
352.875	10.90	8.48	15.66
353.000	10.72	8.39	16.31
353.125	10.80	9.24	15.70
353.250	10.81	9.18	15.36
353.375	11.27	9.51	15.26
353.500	11.77	9.02	14.97
353.625	12.21	10.40	15.04
353.750	12.49	10.88	15.25
353.875	13.05	10.74	15.57
354.000	13.55	12.05	15.34
354.125	14.32	12.30	14.85
354.250	14.99	13.19	15.69
354.375	15.28	13.10	14.26
354.500	14.51	13.38	14.60
354.625	14.55	12.73	13.98
354.750	14.48	13.27	14.55
354.875	14.14	12.44	14.27
355.000	13.79	11.74	14.20
355.125	13.43	11.62	13.63
355.250	13.11	10.92	14.61
355.375	12.75	10.74	14.17
355.500	12.59	10.85	13.98
355.625	12.41	10.47	13.82
355.750	12.34	10.36	13.73
355.875	12.24	10.49	13.14
356.000	12.38	10.41	13.32
356.125	12.43	10.33	13.13
356.250	12.55	10.91	13.20
356.375	12.99	10.92	13.45
356.500	12.84	11.22	13.20
356.625	13.69	12.25	12.40
356.750	13.88	12.23	13.24
356.875	14.65	12.84	13.07
357.000	15.00	13.19	12.88
357.125	15.18	14.09	13.00
357.250	15.12	13.89	12.54
357.375	14.89	13.30	12.94
357.500	14.98	12.92	12.83
357.625	14.25	12.67	12.77
357.750	13.94	12.27	13.83
357.875	13.82	12.19	12.36
358.000	13.54	12.02	12.60
358.125	13.84	11.73	12.50
358.250	13.29	11.55	11.74
358.375	13.35	11.28	12.56
358.500	13.05	11.31	12.55
358.625	12.82	11.09	11.39
358.750	12.80	10.46	12.20
358.875	12.23	10.72	11.68
359.000	12.24	9.87	11.63
359.125	12.04	10.43	11.75
359.250	12.26	10.70	12.00
359.375	12.14	10.09	11.77
359.500	12.47	10.87	11.76
359.625	12.40	10.32	11.81
359.750	12.49	10.65	11.39
359.875	12.03	10.95	11.04
360.000	12.13	10.48	11.05
360.125	12.57	10.66	10.31
360.250	12.68	11.16	11.33
360.375	13.15	11.61	11.02
360.500	13.54	11.49	11.31
360.625	13.77	12.05	10.81
360.750	14.06	12.32	10.34
360.875	14.28	12.49	10.17
361.000	14.48	12.25	10.27
361.125	14.38	12.53	10.57
361.250	13.74	12.50	11.09
361.375	13.80	12.11	10.12
361.500	13.75	11.44	10.05
361.625	13.73	11.92	10.41
361.750	13.48	11.34	10.45
361.875	13.31	11.47	10.34
362.000	13.55	12.07	9.99
362.125	13.50	11.22	9.82
362.250	13.69	12.13	9.38
362.375	13.86	12.14	9.67
362.500	13.66	11.98	9.86
362.625	13.73	12.03	9.41
362.750	13.43	11.86	9.73
362.875	13.85	12.18	9.17
363.000	13.77	12.22	9.41
363.125	13.41	11.52	9.42
363.250	13.49	12.20	9.79
363.375	13.57	12.14	9.08
363.500	13.22	11.27	9.51
363.625	13.24	11.11	8.96
363.750	12.78	11.18	8.61
363.875	12.85	10.81	8.85
364.000	13.10	11.78	8.67
364.125	13.58	12.07	8.11
364.250	14.09	12.42	8.15
364.375	14.40	12.90	7.13
364.500	14.55	12.89	7.95
364.625	14.77	13.77	9.05
364.750	14.96	13.89	8.09
364.875	15.15	14.03	7.71
365.000	15.54	14.44	7.80
365.125	15.75	15.05	5.71
365.250	16.15	15.06	6.23
365.375	15.97	15.42	6.33
365.500	16.01	14.60	6.34
365.625	15.68	14.63	5.98
365.750	15.55	14.10	6.47
365.875	14.85	13.40	6.35
366.000	14.51	13.00	6.82
366.125	13.91	12.28	6.03
366.250	13.57	11.69	6.71
366.375	13.35	11.87	5.42
366.500	13.17	11.66	5.81
366.625	13.11	11.97	5.56
366.750	13.03	11.98	5.05
366.875	13.82	12.62	5.17
367.000	13.94	12.62	4.76
367.125	14.60	13.27	5.35
367.250	14.99	13.64	5.82
367.375	14.89	13.96	5.06
367.500	14.67	13.58	5.03
367.625	14.47	13.38	5.49
367.750	14.85	13.44	4.97
367.875	14.65	13.67	5.15
368.000	14.36	13.42	4.78
368.125	14.45	13.38	4.64
368.250	14.40	12.73	4.93
368.375	14.16	12.88	5.43
368.500	14.18	13.06	5.13
368.625	13.87	12.45	5.08
368.750	13.83	12.61	5.13
368.875	14.10	12.61	4.47
369.000	13.93	12.70	4.81
369.125	14.27	13.30	4.28
369.250	14.42	13.43	4.48
369.375	14.39	12.79	4.51
369.500	14.05	12.88	4.49
369.625	13.91	13.11	4.32
369.750	14.06	12.60	4.15
369.875	14.31	13.08	3.82
370.000	14.57	13.08	4.39
370.125	14.35	13.05	5.23
370.250	14.19	12.81	3.98
370.375	14.07	12.78	4.15
370.500	13.93	12.40	3.95
370.625	13.67	11.54	3.65
370.750	13.17	12.00	3.40
370.875	13.34	11.35	3.96
371.000	14.01	11.92	3.77
371.125	14.02	12.43	4.09
371.250	14.01	12.85	4.10
371.375	14.66	13.16	3.35
371.500	14.83	13.92	3.56
371.625	15.40	14.42	3.89
371.750	15.80	14.57	3.68
371.875	15.98	14.69	3.33
372.000	16.08	15.46	3.64
372.125	16.29	14.87	3.92
372.250	16.06	15.19	3.76
372.375	16.26	15.03	3.62
372.500	16.06	14.60	3.96
372.625	15.80	14.83	3.40
372.750	15.32	14.47	3.04
372.875	14.96	14.04	3.05
373.000	14.79	13.79	3.22
373.125	14.69	13.56	2.89
373.250	14.35	13.06	3.59
373.375	14.37	13.13	3.83
373.500	14.06	12.88	2.80
373.625	14.54	12.77	3.07
373.750	14.58	13.20	3.50
373.875	14.68	13.56	3.87
374.000	14.41	13.35	2.56
374.125	14.46	13.28	3.47
374.250	13.93	12.83	2.97
374.375	13.85	12.15	2.82
374.500	13.68	12.10	3.30
374.625	13.65	11.92	3.03
374.750	13.81	12.34	2.66
374.875	14.11	12.62	2.25
375.000	14.39	13.43	2.49
375.125	14.91	14.27	3.24
375.250	15.32	13.92	2.54
375.375	15.78	14.48	2.67
375.500	15.86	14.64	2.39
375.625	15.85	14.35	2.32
375.750	15.99	14.78	1.35
375.875	15.99	14.79	2.08
376.000	16.76	15.70	1.93
376.125	17.48	16.52	2.71
376.250	17.57	16.81	2.91
376.375	17.34	16.24	2.49
376.500	16.93	16.05	2.76
376.625	16.65	15.62	2.32
376.750	16.33	15.47	2.24
376.875	15.28	14.38	2.23
377.000	15.24	13.86	2.87
377.125	14.69	13.40	2.43
377.250	14.21	12.97	2.11
377.375	13.99	12.60	2.09
377.500	14.08	12.29	1.94
377.625	14.06	12.39	1.82
377.750	14.08	12.51	2.01
377.875	14.19	12.68	1.88
378.000	13.91	12.78	1.76
378.125	13.63	12.51	1.85
378.250	13.76	12.26	1.89
378.375	13.88	12.60	1.74
378.500	14.04	12.50	2.56
378.625	14.21	13.04	1.38
378.750	14.04	12.77	1.20
378.875	14.42	13.10	1.50
379.000	14.70	13.60	.83
379.125	14.96	13.81	1.51
379.250	15.31	14.71	.62
379.375	15.84	15.00	.99
379.500	16.53	15.60	1.78
379.625	17.00	16.22	1.68
379.750	16.90	16.15	1.76
379.875	16.82	15.81	1.47
380.000	16.09	15.93	1.23
380.125	16.25	15.64	.84
380.250	15.99	15.38	.99
380.375	15.96	16.29	.79
380.500	15.73	15.97	.76
380.625	15.97	16.38	.71
380.750	15.37	15.17	.84
380.875	15.13	14.67	1.45
381.000	15.22	14.49	.42
381.125	15.43	14.79	1.15
381.250	15.36	14.67	.63
381.375	15.03	15.25	.42
381.500	14.83	14.73	.35
381.625	14.91	14.76	.34
381.750	15.08	15.24	.61
381.875	15.10	14.58	.73
382.000	15.15	14.99	.78
382.125	15.52	14.81	.48
382.250	14.73	13.77	.78
382.375	13.83	13.70	.48
382.500	14.16	13.56	.88
382.625	14.22	13.96	1.08
382.750	14.30	13.65	.67
382.875	14.70	14.94	.85
383.000	14.44	14.45	1.10
383.125	14.39	13.98	1.17
383.250	14.83	14.88	1.07
383.375	14.62	14.10	.61
383.500	14.58	13.91	.99
383.625	15.27	13.72	.71
383.750	15.72	14.60	.84
383.875	15.23	14.96	.52
384.000	16.04	15.17	1.24
384.125	15.16	14.48	.59
384.250	15.78	14.80	.57
384.375	15.89	15.71	.34
384.500	15.96	15.70	1.31
384.625	16.28	15.51	.80
384.750	17.18	16.21	.91
384.875	16.58	16.91	.79
385.000	15.97	15.55	0.69
385.125	15.28	14.91	.52
385.250	15.68	14.86	.53
385.375	15.97	15.30	.99
385.500	15.60	15.07	1.07
385.625	16.87	16.03	0.91
385.750	16.40	15.90	.90
385.875	15.36	14.96	1.12
386.000	14.80	14.90	1.00
386.125	14.98	14.46	.61
386.250	15.03	14.06	1.33
386.375	14.43	13.49	0.69
386.500	14.30	13.74	.25
386.625	14.66	14.13	.22
386.750	15.11	13.76	.41
386.875	15.16	14.75	.36
387.000	15.06	15.00	.99
387.125	15.98	15.36	.78
387.250	15.58	15.58	.71
387.375	16.22	15.54	.41
387.500	15.89	15.62	.12
387.625	16.08	15.96	.15
387.750	15.91	15.65	.96
387.875	16.24	16.09	.36
388.000	16.07	14.98	.47
388.125	15.49	15.31	.76
388.250	15.60	14.57	.90
388.375	15.30	14.76	.41
388.500	15.29	14.51	1.00
388.625	15.24	14.91	0.72
388.750	15.53	14.45	.68
388.875	16.11	15.45	.99
389.000	16.19	16.01	1.22
389.125	16.86	16.08	0.38
389.250	16.24	15.96	.30
389.375	16.31	15.85	.82
389.500	16.22	15.05	.71
389.625	15.85	15.03	.57
389.750	15.43	14.58	.17
389.875	16.17	15.60	.39
390.000	16.13	14.77	.76
390.125	16.29	15.46	
390.250	16.68	15.25	
390.375	16.26	15.59	
390.500	16.07	15.25	
390.625	15.79	15.21	
390.750	15.93	14.57	
390.875	15.42	14.76	
391.000	15.67	14.67	
391.125	15.94	15.41	
391.250	16.19	15.05	
391.375	15.67	14.94	
391.500	16.73	15.80	
391.625	17.68	16.79	
391.750	18.49	17.50	
391.875	17.17	17.14	
392.000	16.26	15.83	
392.125	16.14	15.18	
392.250	16.51	15.48	
392.375	16.10	14.95	
392.500	16.68	15.53	
392.625	16.22	14.79	
392.750	16.13	15.06	
392.875	15.64	14.96	
393.000	14.66	13.86	
393.125	14.47	13.53	
393.250	13.98	12.81	
393.375	14.57	13.79	
393.500	14.76	13.72	
393.625	15.04	14.33	
393.750	14.53	14.43	
393.875	14.78	14.05	
394.000	14.90	14.49	
394.125	14.53	13.69	
394.250	14.28	13.30	
394.375	14.31	13.61	
394.500	14.07	13.15	
394.625	13.91	12.88	
394.750	15.00	13.89	
394.875	15.62	14.13	
395.000	15.83	15.10	
395.125	15.83	14.98	
395.250	16.27	15.99	
395.375	17.46	15.94	
395.500	16.70	15.62	
395.625	17.21	16.42	
395.750	17.25	16.58	
395.875	17.07	16.60	
396.000	16.52	16.03	
396.125	15.92	15.44	
396.250	16.05	14.89	
396.375	15.10	14.48	
396.500	15.03	14.02	
396.625	14.91	13.09	
396.750	14.53	13.71	
396.875	14.53	13.93	
397.000	15.23	13.51	
397.125	14.29	13.34	
397.250	14.43	13.89	
397.375	14.07	13.17	
397.500	14.88	14.46	
397.625	15.97	15.27	
397.750	16.66	16.13	
397.875	17.32	17.31	
398.000	17.22	17.33	
398.125	17.97	17.71	
398.250	18.39	17.13	
398.375	18.60	17.99	
398.500	17.87	16.56	
398.625	17.27	16.39	
398.750	15.94	15.39	
398.875	15.20	14.20	
399.000	15.15	13.66	
399.125	14.83	14.27	
399.250	14.68	13.17	
399.375	15.41	13.25	
399.500	16.05	14.44	
399.625	15.92	15.48	
399.750	17.30	15.85	
399.875	18.09	17.51	
400.000	18.17	17.91	
400.125	17.89	17.91	
400.250	17.27	16.78	
400.375	17.98	17.08	
400.500	17.61	16.01	
400.625	17.21	16.43	
400.750	17.51	16.30	
400.875	17.44	16.66	
401.000	17.54	16.92	
401.125	17.02	15.86	
401.250	16.92	15.24	
401.375	16.87	15.82	
401.500	16.64	14.97	
401.625	16.15	15.23	
401.750	16.53	15.13	
401.875	15.56	14.85	
402.000	15.35	14.52	
402.125	14.69	13.02	
402.250	14.55	13.49	
402.375	14.71	13.68	
402.500	14.31	12.83	
402.625	13.66	12.37	
402.750	14.02	13.16	
402.875	13.91	13.03	
403.000	13.72	12.85	
403.125	14.17	12.63	
403.250	13.85	12.58	
403.375	14.31	13.62	
403.500	14.26	13.28	
403.625	15.02	13.42	
403.750	15.98	15.11	
403.875	16.54	15.45	
404.000	16.31	15.15	
404.125	15.64	14.74	
404.250	15.28	13.87	
404.375	15.40	13.80	
404.500	14.78	13.39	
404.625	15.40	14.40	
404.750	17.60	16.29	
404.875	15.95	15.34	
405.000	16.99	16.02	
405.125	18.17	17.67	
405.250	16.44	17.02	
405.375	16.42	15.29	
405.500	15.58	14.35	
405.625	14.50	13.05	
405.750	14.72	13.33	
405.875	15.44	13.63	
406.000	14.49	13.25	
406.125	15.36	14.61	
406.250	14.93	13.63	
406.375	13.42	11.96	
406.500	13.62	12.46	
406.625	12.67	11.98	
406.750	13.17	11.87	
406.875	12.85	11.68	
407.000	12.71	11.02	
407.125	12.60	11.68	
407.250	13.09	11.36	
407.375	13.42	11.90	
407.500	13.89	12.23	
407.625	15.06	14.05	
407.750	15.08	13.26	
407.875	16.88	15.77	
408.000	16.03	14.65	
408.125	17.93	16.15	
408.250	18.42	17.77	
408.375	18.71	17.86	
408.500	18.01	16.73	
408.625	17.29	16.21	
408.750	16.47	16.01	
408.875	16.82	15.83	
409.000	15.86	13.82	
409.125	15.37	14.08	
409.250	16.66	15.58	
409.375	16.54	15.05	
409.500	17.27	15.47	
409.625	15.76	14.67	
409.750	15.41	13.72	
409.875	15.17	13.44	
410.000	15.52	14.31	
410.125	17.96	15.82	
410.250	18.08	17.75	

## Footnotes

1 Figures in brackets indicate the literature references at the end of this paper.

2 Trade names used in this paper do not imply recommendation or endorsement by the National Bureau of Standards.

3 1 Torr =133.3 Pa.

4 In computing *ϵ*, the experimental pressures have been converted to the equivalent values at 273 K. To convert to absorption cross sections (cm^2^ molecule^−1^), multiply *ϵ* by 3.72 × 10^−20^.

## 4. Appendix A

Consider a volume, *V*, in which is measured a certain pressure, *P_T_*, of an NO_2_–N_2_O_4_ equilibrium mixture at temperature *T.* The number of nitrogen atoms is *N*= (*V/RT*) (*P*_1_+2*P*_2_) where *P*_1_ and *P*_2_ refer to the pressure of monomer and dimer, respectively. If we follow the procedure utilized in the experiments, the gas is now transferred completely to the cell with volume, *V*′ and subsequently vaporized. A fraction (*f*) of the cell is maintained at a reduced temperature, *T** and the remainder at *T*. The number of nitrogen atoms in the warm fraction is then

$$N=\frac{\left(1-f\right)V\prime }{RT}\left(P_{1}^{\prime }+2P_{2}^{\prime }\right)$$ (1)

while the number in the cold portion is:

$$N=\frac{fV\prime }{RT^{*}}\left(P_{1}^{*}+2P_{2}^{*}\right).$$ (2)

Since the number of N atoms is conserved:

$$\frac{V\prime }{R}\left[\left(\frac{1-f}{T}\right)\left(P_{1}^{\prime }+2P_{2}^{\prime }\right)+\frac{f}{T^{*}}\left(P_{1}^{*}+2P_{2}^{*}\right)\right]=\frac{V}{RT}\left(P_{1}+2P_{2}\right).$$ (3)

If we set (1−*f*)/*T=a* and *f/T* = b*, eq (3) reduces to:

$$aP\prime \left[X_{1}^{\prime }+2\left(1-X_{1}^{\prime }\right)\right]+bP*\left[X_{1}^{*}+2\left(1-X_{1}^{*}\right)\right]=\frac{V}{V\prime }\frac{1}{T}\left(P_{1}+2P_{2}\right)$$ (4)

where *X*_1_ is the mole fraction of NO_2_. Since the volume ratio, *V/V*′ may be measured, the RHS of eq (4) is known. Recognizing that the pressure throughout the cell is constant, *P*′=*P**, and eq (4) may be solved by iteration. An initial estimate is made of the total pressure in the absorption cell and then using the equilibrium constant, *K_p_*, appropriate to the temperature (either room or reduced), a new pressure may be obtained. The process is continued until successive calculations yield similar results. Typical values for our apparatus are *V/V′* = 8.9 and *f*= 0.5.

## 5. Appendix B

Chao et al. [1] calculated the thermodynamic properties of NO_2_ and N_2_O_4_. The method involved a statistical thermodynamic calculation based upon a rigid rotor and harmonic oscillator model. Equilibrium constants were obtained from the thermodynamic functions; from a least squares fit the following empirical relationship was obtained.

$$\log_{10}K_{\text{eq}}=0.3199+2.945\times 10^{-5}T-2768.85/T+5.847484\log T-7.89560\left[\left(1+100/T\right)\log\left(1+T/100\right)\right]$$ (1)

Chao et al., note the standard deviation between log *K* calculated from (1) versus that from thermodynamic functions is 0.00055, and agreement between log *K* from (1) and experimental measurements is excellent.

The equilibrium constants used here have been compared with those derivable from the molecular parameters used in the JANAF Thermochemical Tables.^5^ Chao’s expression (1) yields *K_p_* uniformly 4 percent higher than the JANAF value. *K_p_* from (1) is also in good agreement with the experimental *K_p_* s reported by Vosper,^6^ agreeing within 4 percent below 290 K. In both cases the agreement is well within the accuracy of the available thermochemical and molecular data.
